# A novel peptide antagonist of the human growth hormone receptor

**DOI:** 10.1016/j.jbc.2021.100588

**Published:** 2021-03-24

**Authors:** Reetobrata Basu, Khairun Nahar, Prateek Kulkarni, Olivia Kerekes, Maya Sattler, Zachary Hall, Sebastian Neggers, Justin M. Holub, John J. Kopchick

**Affiliations:** 1 Heritage College of Osteopathic Medicine, Ohio University, Athens, Ohio, USA; 2 Edison Biotechnology Institute, Ohio University, Athens, Ohio, USA; 3 Department of Chemistry and Biochemistry, Ohio University, Athens, Ohio, USA; 4 Molecular and Cellular Biology Program, Ohio University, Athens, Ohio, USA; 5 Honors Tutorial College, Ohio University, Athens, Ohio, USA; 6 Department of Medicine, Endocrinology Section, Erasmus University Medical Centre, Rotterdam, the Netherlands

**Keywords:** growth hormone, GH receptor, peptide antagonist, peptide mimetic, site 1-binding helix, ACN, acetonitrile, bGH, bovine GH, CD, circular dichroism, GH, growth hormone, hGH, human growth hormone, hGHR, human growth hormone receptor, hPRL, human prolactin, hPRLR, human prolactin receptor, IGF-1, insulin-like growth factor 1, LS, Laron Syndrome, NMP, N-methyl-2-pyrrolidone, PBS, phosphate buffered saline, PPI, protein–protein interaction, PRL, prolactin, S1H, *s*ite 1-binding helix, TFA, trifluoroacetic acid, TFE, 2,2,2-trifluoroethanol

## Abstract

Excess circulating human growth hormone (hGH) *in vivo* is linked to metabolic and growth disorders such as cancer, diabetes, and acromegaly. Consequently, there is considerable interest in developing antagonists of hGH action. Here, we present the design, synthesis, and characterization of a 16-residue peptide (*s*ite 1-binding *h*elix [S1H]) that inhibits hGH-mediated STAT5 phosphorylation in cultured cells. S1H was designed as a direct sequence mimetic of the site 1 mini-helix (residues 36–51) of wild-type hGH and acts by inhibiting the interaction of hGH with the human growth hormone receptor (hGHR). *In vitro* studies indicated that S1H is stable in human serum and can adopt an α-helix in solution. Our results also show that S1H mitigates phosphorylation of STAT5 in cells co-treated with hGH, reducing intracellular STAT5 phosphorylation levels to those observed in untreated controls. Furthermore, S1H was found to attenuate the activity of the hGHR and the human prolactin receptor, suggesting that this peptide acts as an antagonist of both lactogenic and somatotrophic hGH actions. Finally, we used alanine scanning to determine how discrete amino acids within the S1H sequence contribute to its structural organization and biological activity. We observed a strong correlation between helical propensity and inhibitory effect, indicating that S1H-mediated antagonism of the hGHR is largely dependent on the ability for S1H to adopt an α-helix. Taken together, these results show that S1H not only acts as a novel peptide-based antagonist of the hGHR but can also be applied as a chemical tool to study the molecular nature of hGH–hGHR interactions.

Human growth hormone (hGH) is a 191-amino acid peptide hormone that acts as a key stimulator of cell growth, replication, and regeneration (1, 2, 3, 4). hGH is secreted from anterior pituitary somatotrophs in a pulsatile manner under the positive regulation of hypothalamic growth hormone (GH)-releasing hormone and gastric ghrelin and the negative regulation of endocrine insulin-like growth factor 1 (IGF-1) and hypothalamic somatostatin (3). In addition to the pituitary gland, hGH can be released peripherally by immune, neural, reproductive, alimentary, and respiratory tissues and in the integumentary, muscular, skeletal, and cardiovascular systems (5, 6, 7, 8, 9). Following entry into the bloodstream, hGH targets the extracellular domain of the human growth hormone receptor (hGHR) on the surface of cells that express the hGHR, including adipocytes, lymphocytes, and hepatocytes (10). The hGH–hGHR binding event induces a conformational change in the hGHR, ultimately activating kinases that are associated with the intracellular portion of the receptor. Such kinases then propagate downstream signaling cascades that lead to the expression of growth-promoting genes (11, 12, 13).

The biological effects of hGH are tissue specific and can be divergent depending on which cells are being targeted. For instance, hGH stimulates anabolic effects in muscle and bone and catabolic effects in adipose tissue (14, 15, 16). Contrary to these beneficial effects, aberrant or dysfunctional hGH activity can promote the onset of disease. For example, elevated hGH levels in the bloodstream inhibit insulin action, leading to insulin resistance and diabetes (17, 18, 19). Moreover, excess circulating hGH caused by hypersecreting pituitary adenomas results in a condition known as acromegaly (20). If left untreated, acromegaly causes severe physiological complications including gigantism, fluid retention, disfigurement, diabetes mellitus, blindness, cardiomyopathy, organ failure, hypertension, and premature death (20, 21, 22). In contrast, organic or idiopathic hGH deficiencies lead to reduced growth rates, impaired bone metabolism, and increased adiposity (23, 24). Apart from issues arising from excess or reduced production of hGH, genetic defects in the hGHR result in congenital hGH insensitivity. For example, inactivating mutations in the hGHR can cause Laron Syndrome (LS), a genetic defect in which patients are insensitive or resistant to hGH action (25, 26). Individuals with LS often present with reduced serum levels of IGF-1, increased levels of hGH, diminished longitudinal growth, and increased fat deposition (26, 27, 28). Remarkably, however, LS patients exhibit enhanced resistance to certain diseases such as diabetes and cancer (29).

A comprehensive understanding of how hGH signals through the hGHR is an evolving process. To gain insight into the physiological nature of GH action, researchers have developed rodent models that successfully recapitulate conditions of aberrant hGH activity observed in humans (2, 4, 30, 31). Such models are valuable for studying GH biology *in vivo* and have demonstrated that antagonizing the GHR can increase lifespan, decrease cancer incidence, lower insulin resistance, and reduce the loss of cognition (32). Mice with targeted global disruption of the GHR gene (GHRKO mice) are excellent models of LS, presenting an obese, growth-retarded phenotype with low levels of IGF-1 and insulin, high levels of GH, and enhanced resistance to diabetes and cancer (2, 33, 34, 35, 36, 37, 38, 39, 40, 41, 42). Notably, GHRKO mice can live for up to 5 years and are officially recognized as the longest-lived laboratory mouse used in research (2). Given that many physiological benefits are correlated with diminished GHR activity, it is perhaps not surprising that considerable effort has been devoted to developing antagonists of the hGH–hGHR interaction.

The hGHR is a 638-amino acid class I cytokine receptor that is involved in the regulation of important physiological processes, including postnatal longitudinal growth, metabolism, organ development, and sexual maturation. Structurally, the hGHR is a single-pass transmembrane protein that contains three discrete domains: an extracellular domain (residues 19–262), a transmembrane domain (residues 263–288), and an intracellular domain (residues 289–638) (10, 43). In the classic model of hGH–hGHR signaling, hGH binds to the extracellular domain of the hGHR homodimer and causes the receptor to rotate approximately 42 degrees relative to the transmembrane axis (44, 45). The hGH–hGHR binding event leads to the transactivation of protein kinases such as JAK2, SRC, and LYN that are associated with the intracellular domain of the hGHR (46, 47, 48). Downstream signaling through the hGHR is complex and involves numerous pleiotropic cytoplasmic signaling cascades. For instance, binding of hGH to the hGHR activates JAK2 and LYN, which in turn activate the STAT1, STAT3, STAT5, Grb2-SOS-MEK-ERK, PI3K-AKT-mToR, and PKC/Ca^2+^ pathways (10, 47). The hGHR is widely expressed on cells of various tissues including liver, fat, bone, muscle, kidney, brain, and skin (32, 49). Furthermore, it has been documented that certain cancers overexpress the hGHR and produce excess levels of hGH, creating a favorable autocrine and paracrine microenvironment for the growing tumor (3, 32).

Mature 22-kDa hGH is encoded by the *GH1* gene located on chromosome 5. A less abundant 20-kDa hGH isoform is expressed following alternative splicing of the second intron within the *GH1* precursor mRNA and does not encode amino acids 32 to 46 (50, 51). Mammalian GHs are highly homologous (70–80%) across species, with notable similarities in their amino acid sequences (52). The first three-dimensional structure of a mammalian GH was reported in 1987, when a genetically engineered variant of porcine GH was crystallized and resolved by X-ray diffraction at a resolution of 2.8 Å (53). This study demonstrated that porcine GH is predominantly helical, consisting of four antiparallel α-helices arranged in a twisted helical bundle (53). The co-crystal structure of the hGH–hGHR complex was first reported in 1992 (54) and confirmed that the binding stoichiometry between hGH and the hGHR occurs at a ratio of 1:2 (54, 55, 56). These structural studies have provided insights into the molecular nature of the hGH–hGHR interaction, revealing that hGH has four distinct α-helices that are closely packed in an up-up-down-down orientation when bound to the hGHR. The co-crystal structure also showed that hGH contains three largely unstructured regions that are classified as a “large loop” (residues 33–75), a “smaller loop” (residues 129–154), and a “small loop located at the C terminus”. A total of 103 amino acids (54%) of hGH are contained within the four antiparallel α-helices and are ordered as follows: helix I (residues 9–34), helix II (residues 72–92), helix III (residues 106–128), and helix IV (residues 155–184). Additionally, the “large loop” between the α-helices I and II (residues 33–75) contains two “mini-helices” comprised of residues 38 to 47 and 64 to 69. In the hGH–hGHR complex, one hGH molecule binds asymmetrically to a preformed hGHR homodimer through two distinct binding sites on the hGH (site 1 and site 2). When bound to the dimeric receptor, site 1 of the hGH interacts with the first hGHR monomer via helix I, helix IV and the two mini-helices located within the “large loop”. Site 2 of the hGH interacts with the second hGHR monomer, primarily through the N terminus of helix I and the central portion of helix III. We have shown that glycine 120 (glycine 119 of bovine GH [bGH]) in helix 3 a crucial amino acid that promotes functional dimerization (*vide infra*).

A detailed analysis of the bGH–GHR interaction indicated that amino acid residues 109 to 126 within helix III of bGH are arranged in an amphipathic helix (57, 58, 59). However, the presence of amino acids E117, G119, and A122 caused the amphipathicity of this helix to be somewhat “imperfect” (59, 60). Notably, this information was used to develop a highly potent antagonist of the hGHR. To establish a more “perfect” amphipathic configuration and facilitate binding of the ligand, amino acids contained within helix III were substituted individually and resulted in bGH analogs that displayed novel inhibitory effects (59, 61). For example, a G119R substitution in bGH (corresponding to G120R in hGH) produced a molecule that acted as an antagonist of mouse GH action *in vitro* and *in vivo* (61). In addition, transgenic mice expressing a G119R GH analog displayed low levels of IGF-1 and a growth-retarded phenotype (60). The importance of hGH site 2 for binding the GHR was confirmed in subsequent studies by the discovery of a new hGHR antagonist containing a G120K mutation (62, 63). This analog binds effectively to the first hGHR subunit through site 1 and improperly to the second hGHR subunit, resulting in a nonfunctional hGH–hGHR complex. This novel hGHR antagonist was eventually developed into the FDA-approved drug, Somavert (pegvisomant for injection), a PEGylated hGH analog that contains nine amino acid substitutions, including G120K (64). Pegvisomant is highly efficacious at attenuating hGHR activation and normalizing serum IGF1 levels in patients with elevated GH (62, 64) and is currently the only clinically approved GHR antagonist marketed worldwide to treat patients with acromegaly (2, 62). Pegvisomant is administered to individuals through daily, subcutaneous injections (2, 65, 66).

However, despite its clinical efficacy, there is growing interest among clinicians and medicinal chemists to improve on pegvisomant by developing longer-acting hGHR antagonists that can be used to treat a range of hGH-mediated diseases (3). Furthermore, pegvisomant is classified as a recombinant protein drug that mimics the full-length hGH molecule. Recombinant protein drugs are often expensive to manufacture and can be beset by end-product heterogeneity (67, 68). In an effort to develop a second-generation hGHR antagonist that could circumvent these issues, we focused on developing a short, helical peptide that mimics a region within site 1 of hGH that is critical for hGHR recognition. We reasoned that site 1-binding interactions between hGH and hGHR could be inhibited using a peptide mimetic that was based on the mini-helix (residues 38–47) found in the “large loop” of hGH. It has been suggested that synthetic peptides offer a viable alternative to larger proteins for targeting therapeutically relevant protein–protein interactions (PPIs). Indeed, peptides are considered advantageous in drug development because they occupy a unique “middle space” that combines desirable attributes of small molecules (synthetic tractability) and large proteins (epitope targeting) into a single construct. Additionally, peptides under 50 amino acids in length can be generated through convenient chemical synthesis procedures in the laboratory, affording researchers exquisite control over the sequence, homogeneity, and purity of the final product. Moreover, peptides provide versatile scaffolds from which to design effective inhibitors of PPIs because they can be engineered to fold into stable three-dimensional structures that mimic protein interaction domains [reviewed in (69)]. Synthetic peptides that fold into stable α-helices, β-strands, and loop structures (70, 71, 72, 73, 74) have been used to successfully target protein–protein and protein–DNA interactions (75, 76, 77), with several of these constructs entering clinical trials (78).

In this report, we outline the design, synthesis, and characterization of a 16-residue peptide that mimics the mini-helix contained within the “large loop” of hGH site 1. This peptide, designated S1H for Site 1-binding Helix, is a near-perfect sequence mimetic of hGH between residues 36 to 51. Based on previous X-ray crystallography (79) and *in vitro* binding data (80), this region of hGH is thought to be crucial for facilitating favorable hGH–hGHR interactions. We therefore reasoned that a synthetic peptide mimic of this helix could be used as an effective antagonist of hGHR activity. Using rational design, we developed S1H as a potent hGHR antagonist and showed that it inhibits hGH-mediated phosphorylation of STAT5 in cultured cells that express the hGHR. Furthermore, we generated a library of S1H sequence analogs to identify which residues within the site 1 mini-helix are important for the structural organization and biological activity of the S1H peptide. Importantly, these studies demonstrate the utility of employing a short peptide not only as an antagonist of a therapeutically relevant PPI but also as an effective chemical tool to study the molecular nature of hGH–hGHR interactions.

## Results

### Rational design of the S1H peptide

**Figure 1:**
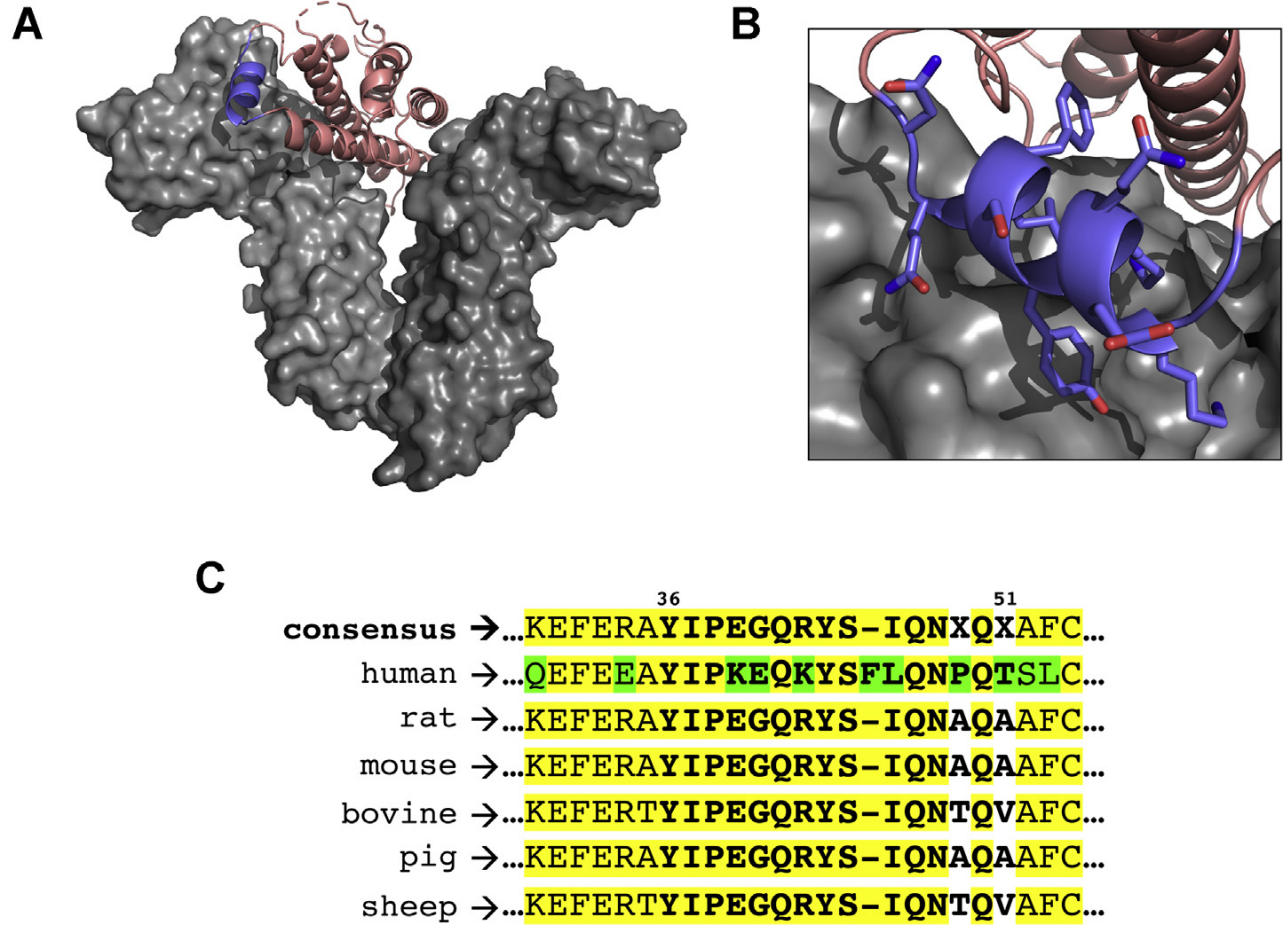
**Rational design of the S1H peptide.***A,* three-dimensional crystal structure of hGH bound to homodimeric hGHR. hGH (*salmon*) is shown as a ribbon diagram bound to the hGHR (*gray*); the site 1 mini-helix of hGH is colored purple. *B*, zoomed image of the site 1 mini-helix bound to the hGHR. Side chains of residues 38 to 47 are shown as stick representations. Structures adapted from PDB ID: 1HWG and rendered using PyMol Molecular Graphics System software (Schrödinger, LLC). *C*, amino acid sequence alignment of GH proteins from human, rat, mouse, cow, pig and sheep indicating conserved (*yellow*) and variable (*green*) amino acid residues. The S1H peptide sequence is shown in *bold*; alignment rendered using Multiple Sequence Alignment Tool (NCBI). GH, growth hormone; hGH, human growth hormone; hGHR, human growth hormone receptor.

Efforts to develop a novel peptide-based antagonist of the hGHR were initiated with a comprehensive analysis of the hGH–hGHR interaction (Fig. 1*A*). The three-dimensional structure of the hGH–hGHR complex (79) shows that hGH binds two separate hGHR subunits through two distal sites on hGH (site 1 and site 2). Dynamic modeling simulations of the hGH–hGHR binding event have indicated that hGH first makes contact with one hGHR monomer via site 1 before binding to the second hGHR monomer via site 2 (80). To reduce the influence of induced or additive binding effects resulting from the preassociation of native hGH to the hGHR, we focused our attention on designing a peptide-based antagonist that would target the incipient (site 1) interaction. At this point, several regions within the hGH–hGHR interaction interface were considered for targeted inhibition. We initially noticed that hGH makes contact with the hGHR on the N-terminal side of the second mini-helix (residues 64–69) located within the “large loop” of hGH. However, this region of hGH is inherently unstructured and was not expected to provide a large enough surface area for effective inhibition. We also noted that helix 4 (residues 168–179) of hGH makes direct contact with the first hGHR monomer at a site that is proximal to the hGHR dimerization interface. Targeting this site for inhibition was also deemed suboptimal because the inhibitor peptide would have to compete sterically with amino acid residues of the hGHR that are involved in receptor dimerization. Following this initial assessment, we speculated that the first mini-helix of the hGH “large loop” (residues 38–47) binds the hGHR at a site with high potential for peptide-based inhibition (Fig. 1*B*). Our hypothesis was based on the following considerations: (1) this specific interaction occurs within site 1 of hGH; (2) the binding site is distal from the hGHR dimerization interface; (3) the interaction is comprised of a relatively large binding surface; and (4) the hGHR-binding domain of hGH is helical at this site. We next moved to design a peptide that would act as a direct sequence mimetic of the hGH mini-helix (residues 38–47) located within the “large loop” between helices I and II. The S1H peptide corresponds to residues 36 to 51 of full-length hGH and includes the entire helical recognition motif flanked on each side by a short sequence of amino acids (YIPKEQKYSFLQNPQT). These flanking residues were included to enhance specific recognition between the peptide substrate and its target protein (81). Interestingly, amino acid sequence alignments of GHs from different vertebrates revealed multiple variations in this region (Fig. 1*C*). According to these sequences, K39, E40, K42, F45, L46, and P49 residues are unique to hGH, whereas Q41, Y43, S44, Q47, N48, and Q50 are well-conserved among various species. In addition, the 20 kDa hGH isoform (*vide supra*) differs from the 22 kDa WT hGH because of a splice variation that deletes 15 amino acids between residues 32 to 46 (50). Notably, this region overlaps significantly with site 1 of hGH and the sequence of the S1H peptide. The 20 kDa hGH isoform has been reported to have a 10-fold lower binding affinity for the hGHR compared with the 22 kDa hGH (82), which hints strongly at the presumed ability for S1H peptides to antagonize the hGH–hGHR interaction.

### Structural characterization of the S1H peptide

**Figure 2:**
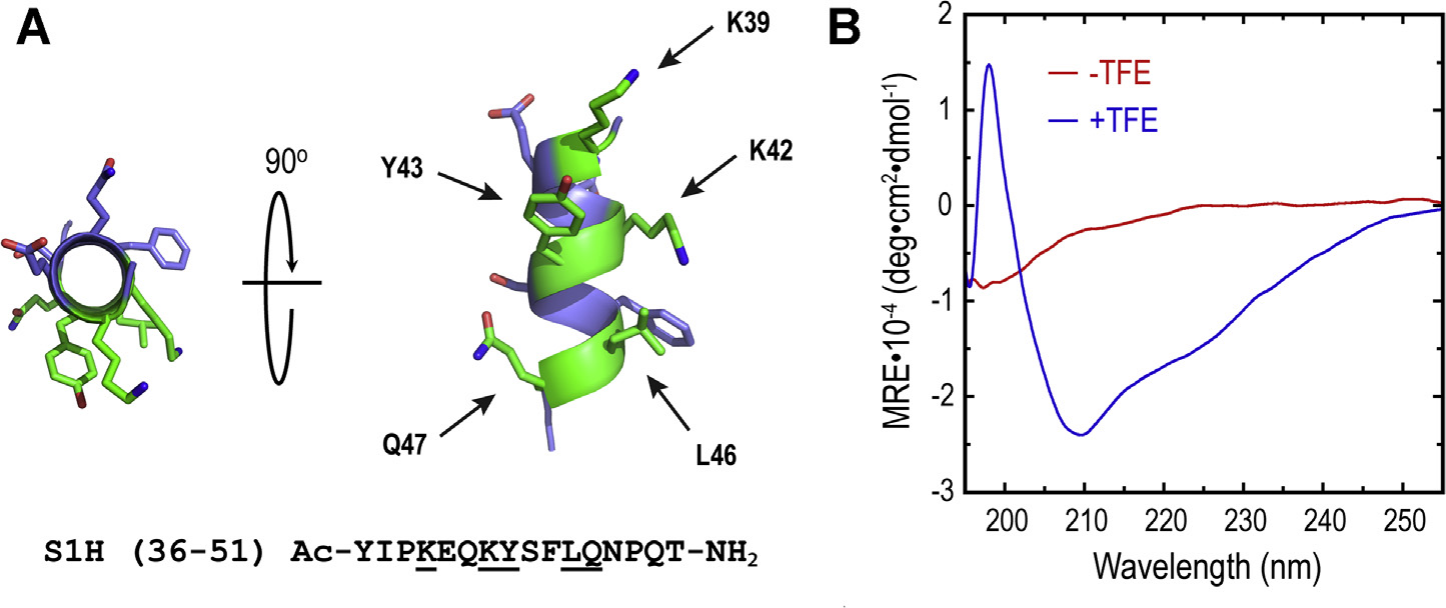
**Structural characterization of S1H peptide.***A,* ribbon diagram of S1H peptide helix. Residues that make contact with the hGHR are colored *green* and indicated with *arrows*. Residues colored *blue* point away from the interface and are not thought to make contact with the hGHR. Primary sequence of S1H is shown below the ribbon diagram; residues that make contact with the hGHR are underlined. N-terminus is shown capped with an acetyl group (Ac); C terminus is an amide (-NH_2_). S1H helix exported from PDB ID: 1HWG. Figure rendered in PyMol. *B*, wavelength-dependent circular dichroism spectra of S1H peptide (20 μM in solution). *Red* spectrum shows S1H in PBS, and *blue* spectrum represents S1H in PBS supplemented with 30% (v/v) TFE. All spectra were collected at 25 °C. hGHR, human growth hormone receptor; PBS, phosphate buffered saline; TFE, 2,2,2-trifluoroethanol.

As mentioned above, the S1H peptide was designed to mimic a helical region of hGH that interacts with the hGHR over a modestly large surface area. According to the hGH–hGHR co-crystal structure, this segment contains at least five residues (K39, K42 Y43, L46, and Q47) that make putative contacts with the surface of the receptor (Fig. 2*A*). At this site, the binding between hGH and the hGHR is believed to be driven by a combination of hydrogen bonding, hydrophobic effects, and electrostatic interactions (Fig. S1). Moreover, because this region of hGH is helical when bound to the hGHR, we speculated that S1H would need to possess inherent helical propensity to elicit favorable interactions with the receptor. Following synthesis and characterization of the S1H peptide (see Materials and Methods, Fig. S2), we evaluated the solution-phase structure of S1H using wavelength-dependent circular dichroism (10.13039/100011639CD) spectropolarimetry (Fig. 2*B*). Far-UV CD measurements were collected from S1H peptide samples (20 μM) dissolved in phosphate buffered saline (PBS) at 25 °C and plotted as mean residue ellipticity *versus* wavelength (195 nm to 255 nm). S1H was found to contain a slight shoulder around 215 nm and a minimum at 198 nm in PBS, indicating that this peptide is inherently unstructured under these conditions. It is worth noting that unless endowed with some structure-inducing linkage or sidechain complex, peptide oligomers comprised of less than 20 amino acids often adopt random coils in solution (83, 84). We next evaluated the helical propensity of S1H by dissolving the peptide (20 μM) in PBS supplemented with 30% (v/v) 2,2,2-trifluoroethanol (TFE) and assessing its structure using wavelength-dependent CD spectropolarimetry as described above. TFE is a structure-inducing co-solvent that can be used to evaluate the propensity of short peptide oligomers to fold in solution (85). Results from these experiments showed that TFE had a dramatic effect on the solution-phase structure of the S1H peptide (Fig. 2*B*). Here, the CD spectrum of S1H showed a shoulder at 225 nm, a minimum at 210 nm and a maximum at 198 nm. This specific CD signature indicates that S1H undergoes a transition from a random coil to a predominantly folded structure following the addition of TFE. Helical populations for each sample were estimated using the mean residue ellipticity at 222 nm, while taking into account the peptide length (see Materials and Methods) (86). From these calculations, it was determined that S1H dissolved in PBS alone was 1.1% helical, whereas S1H incubated in PBS supplemented with TFE showed 48.2% helicity. These results demonstrate that a significant proportion of S1H peptide is capable of adopting a helical fold in the presence of structure-inducing co-solvents. Perhaps more importantly, however, the inherent helical propensity of S1H suggests that this peptide has the ability to fold into a structural configuration that can mimic the site 1 mini-helix of hGH when targeting the hGHR.

### Stability profiles of S1H, hGH, and pegvisomant in human serum

**Figure 3:**
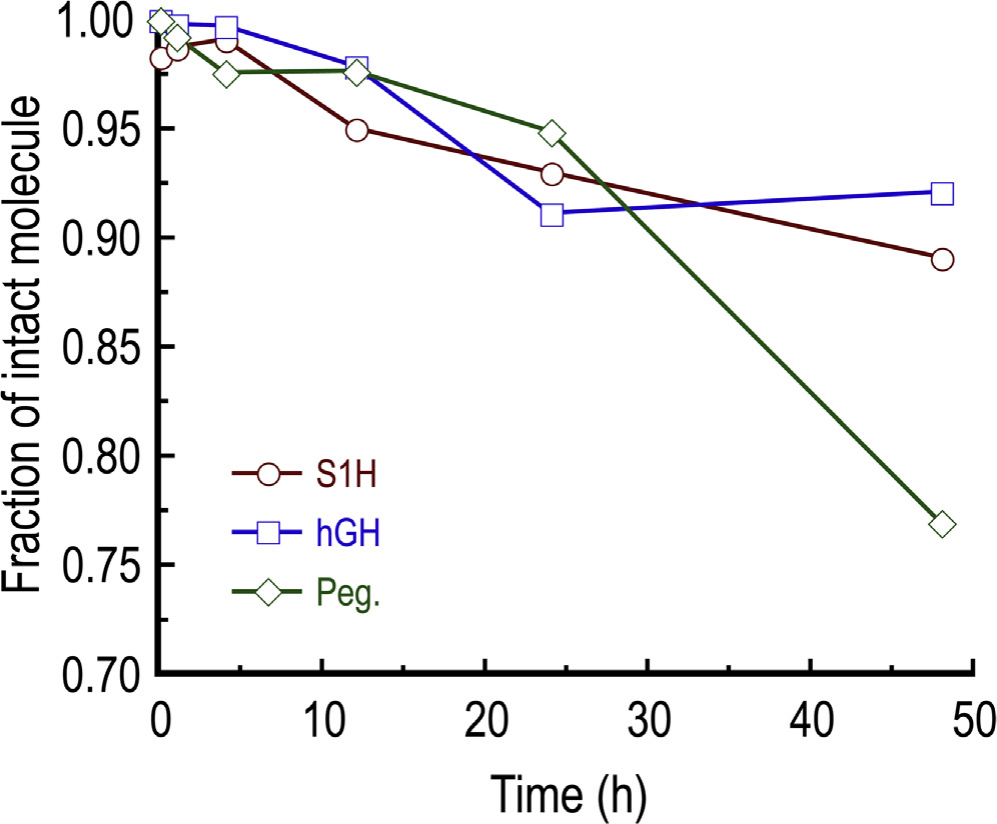
**Quantification of peptide and protein stability (reported as fraction of intact molecule) following incubation in 25% (v/v) human AB serum for 0 to 48 h.** Fractions of intact S1H were determined from analytical HPLC chromatograms; fractions of intact hGH and pegvisomant (Peg.) were determined using *western blots*. See Experimental procedures and supporting information for experimental details. hGH, human growth hormone.

Peptide and protein hormones released into the bloodstream often have short half-lives due in part to relatively high concentrations of proteases in the serum (87, 88). Indeed, proteolytic susceptibility is often a major limitation that hinders the therapeutic efficacy of peptide- and protein-based drugs (89). We were therefore interested in testing the stability of S1H in a serum-rich microenvironment. To assess this, we incubated S1H peptides in RPMI media supplemented with 25% (v/v) human AB serum for up to 48 h at 37 °C and quantified the fraction of intact peptide using reversed-phase HPLC (Figure 3 and Fig. S3). We observed that the S1H peptide was not significantly degraded under these conditions, with >89% of the peptide remaining intact after 48 h incubation. This indicates that the S1H peptide is inherently stable in a serum-rich microenvironment and should remain intact as it is delivered to cultured cells (*vide infra*). As a positive control for degradation of the peptide, we incubated S1H with trypsin at 37 °C and similarly monitored its degradation by reversed-phase HPLC (Fig. S4A). Under these conditions, it was shown that the S1H peptide was rapidly degraded to three major products (likely because of to the presence of two lysines within the S1H sequence), with no intact peptide detected after 15 min incubation. To gain further insight into how the stability of S1H compares to that of hGH and pegvisomant, we tested the stability of the recombinant proteins under similar conditions. For these experiments, hGH and pegvisomant were dissolved in RPMI media supplemented with 25% (v/v) human serum and incubated at 37 °C for up to 48 h. Following incubation, the proteins were separated by SDS-PAGE, and the extent of degradation was assessed by Western blotting (Figure 3, Fig. S5). Interestingly, it was determined that hGH was more stable than either S1H or pegvisomant under these conditions, with >92% of the protein remaining intact following 48 h incubation. On the other hand, pegvisomant was the least stable in human serum, with only 76% of the protein remaining after 48 h. As positive controls for protein degradation, hGH and pegvisomant were incubated with trypsin at 37 °C for up to 8 h and monitored by HPLC and Western blotting (Figs. S4, *B* and *C*, and S5). Here, it was observed that hGH was fully degraded after 8 h incubation, whereas pegvisomant only took 4 h to be digested under similar conditions. This result was somewhat surprising, as pegvisomant contains multiple PEGylations that are believed to increase its half-life *in vivo* compared with hGH (90). It could be speculated that the higher degree of degradation seen with pegvisomant is attributed to the loss of PEG conjugates during the incubation or comparatively enhanced binding to serum proteins (91, 92).

### S1H attenuates hGH-mediated STAT5 phosphorylation in human and mouse cells

**Figure 4:**
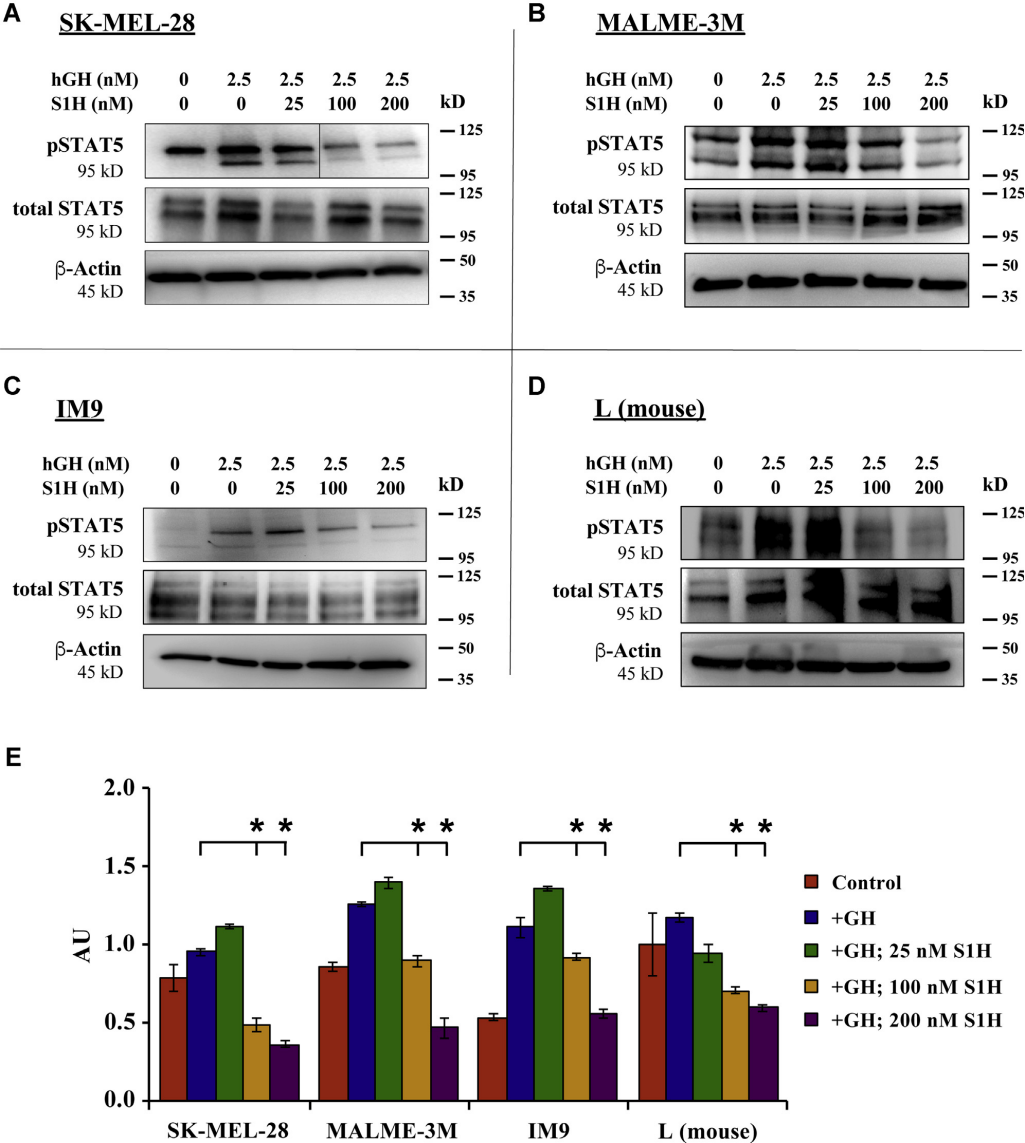
**S1H inhibits hGH-mediated STAT5 phosphorylation in cultured cells.** For these experiments, cells were treated with S1H peptide (0–200 nM) in the presence of 2.5 nM hGH for 20 min. Total protein was then extracted from the cells, and intracellular concentrations of pSTAT5 were evaluated by Western blot. Representative Western blot images (n = 3) are presented for cell lines expressing high levels of GHR. *A*, SK-MEL-28 cells; *B* MALME-3M cells; *C*, IM9 cells; and *D*, L (mouse fibroblast) cells. Size marker (kD) is shown to the right of the images. *E*, relative ratio of pSTAT5 to total STAT5 quantified from densitometry analysis of *western blots* shown in panels *A–D*. *Error bars* are standard deviation; ∗*p* < 0.05. AU, absorbance units; hGH, human growth hormone; GHR, growth hormone receptor.

A major downstream effect of hGH-mediated activation of the hGHR is the phosphorylation of intracellular transcription factors, including STAT5A and STAT5B (93, 94). In this signaling cascade, binding of hGH to hGHR leads to transactivation of hGHR-associated JAK2, which phosphorylates the intracellular domain of hGHR at five tyrosine residues: Y332, Y487, Y534, Y566, and Y627 (95). STAT5 then docks via its SH2 domains to the phosphorylated hGHR and is subsequently phosphorylated by JAK2 (10, 11). The phosphorylation of STAT5 (pSTAT5) can be observed just minutes after adding hGH to cultured cells, making intracellular pSTAT5 quantitation a convenient metric of hGHR activation. Inhibitors of hGHR action, such as pegvisomant and FGF21, have been shown to block hGH-mediated pSTAT5 in cell lines that express high levels of hGHR (96). Here, we used intracellular pSTAT5 levels to quantify S1H-mediated antagonism of the hGHR in cultured human melanoma cells (SK-MEL-28 and MALME-3M), human lymphoblasts (IM9), and mouse (L) fibroblasts. The human cells used in these studies were previously reported to express high levels of endogenous hGHR (48, 97), whereas the mouse L fibroblasts express high levels of mouse GHR (98). To determine whether S1H could inhibit hGH-mediated pSTAT5, we co-treated SK-MEL-28 cells, MALME-3M cells, IM9 cells, and mouse L fibroblasts with S1H and hGH and evaluated pSTAT5 levels via Western blotting (Fig. 4). As expected, the addition of 2.5 nM (50 ng/ml) hGH showed significant upregulation of pSTAT5 within 20 min across all cell lines tested. It was also observed that cells co-treated with S1H and hGH displayed significantly lower levels of pSTAT5 compared with cells treated with hGH alone. This inhibitory effect was dose dependent, with hGH-mediated pSTAT5 levels being suppressed by 46% and 67%, respectively, in SK-MEL-28 cells treated with 100 nM or 200 nM S1H (Fig. 4, *A* and *E*). Similar treatments in MALME-3M cells indicated that S1H suppresses pSTAT5 levels by 56% at concentrations of 200 nM in this cell line (Fig. 4, *B* and *E*). Significant degrees of hGHR antagonism were also observed in IM9 and L (mouse fibroblast) cells, with S1H suppressing hGH-mediated pSTAT5 by 67% and 36%, respectively, at 200 nM (Fig. 4, *C–E*). To determine whether S1H can act as a hGHR agonist, we treated SK-MEL-28, MALME-3M, and IM9 cells with 25 nM or 100 nM S1H for 20 min and quantified intracellular pSTAT5 levels by Western blot (Fig. S6). It was observed here that S1H did not increase pSTAT5 concentrations above background (untreated) levels in any cell line tested. Taken together, these results indicate that S1H is able to inhibit the hGH-mediated phosphorylation of STAT5 in cultured cells by acting as an antagonist of the hGHR.

### S1H attenuates activation of hGHR and human prolactin receptor in cultured cells

**Figure 5:**
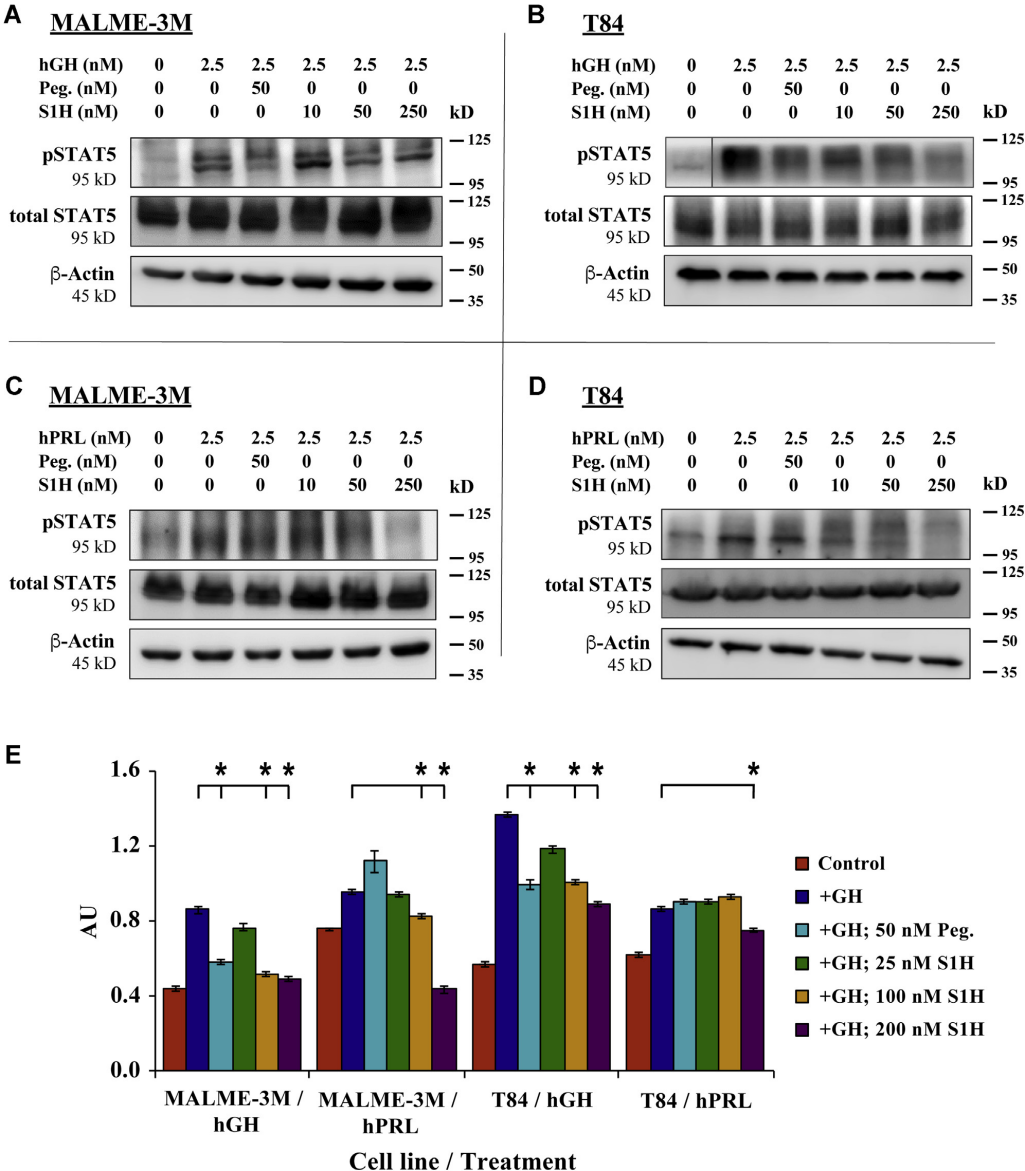
**S1H attenuates hGHR and hPRLR activation in cultured cells**. For these experiments, cells were treated with either hGH or hPRL for 20 min in the presence or absence of pegvisomant or S1H. Total protein was then extracted, and intracellular concentrations of pSTAT5 and total STAT5 were evaluated by Western blot. *A*, MALME-3M cells treated with hGH; *B*, T84 cells treated with hGH; *C*, MALME-3M cells treated with hPRL; *D*, T84 cells treated with hPRL. Size marker (kD) is shown to the right of the images. *E*, relative ratio of pSTAT5 to total STAT5 quantified from densitometry analysis of *western blots* shown in panels *A–D*. *Error bars* are standard deviation. ∗*p* < 0.05. AU, absorbance units; hGH, human growth hormone; hGHR, human growth hormone receptor; hPRLR, human prolactin receptor; hPRL, human prolactin.

Growth hormone, prolactin (PRL), and placental lactogen are all members of the class-I cytokine superfamily. Incidentally, all three cytokines are capable of binding and activating the human prolactin receptor (hPRLR), which leads to phosphorylation and activation of STAT5 (99). Owing to this broad-spectrum activation profile, the mechanistic design of hGHR and hPRLR antagonists often overlap (100). We were therefore interested in determining whether the S1H peptide could interfere with hGH-induced activation of the hPRLR. To answer this question, we treated MALME-3M (human melanoma) and T84 (human colon cancer) cells with either 2.5 nM hGH or hPRL in the absence or presence of S1H and quantified the level of pSTAT5 by Western blotting (Fig. 5). These cell lines were selected for this study based on the following criteria: MALME-3M cells express high levels of hGHR relative to hPRLR (>100:1), whereas T84 cells express high levels of hPRLR relative to hGHR (>1300:1) (101). This allowed us to study whether S1H was able to selectively attenuate hPRLR activation without significant influence of hGHR-mediated signaling. Here, we observed that S1H could suppress hGH-induced pSTAT5 in MALME-3M cells in a dose-dependent manner and demonstrated greater efficacy for hGHR antagonism than pegvisomant. More specifically, we found that 50 nM S1H treatments inhibited pSTAT5 production by 40% compared with cells treated with hGH alone, whereas treatment with 50 nM pegvisomant inhibited STAT5 inhibition by 33% under the same conditions (Fig. 5, *A* and *E*). Both S1H and pegvisomant suppressed the hGH-mediated phosphorylation of STAT5 by similar amounts (27%) at 50 nM in T84 cells (Fig. 5, *B* and *E*). We also observed a clear dose-dependence in S1H-mediated inhibition of pSTAT5 in T84 cells that had been co-treated with hGH. Finally, we observed that unlike pegvisomant, S1H inhibited hPRL-induced pSTAT5 by 49% in MALME-3M cells and 13% in T84 cells (Fig. 5, *C–E*). This result strongly suggests that the S1H peptide can impede the association of hPRL with hPRLR and mitigate downstream signaling cascades that are initiated by this interaction. Furthermore, these findings indicate that despite being designed specifically as an antagonist of the hGH–hGHR interaction, the S1H peptide may have broader inhibitory effects, namely, antagonizing the PRL/PLRR interaction.

### Structural analysis of S1H peptide sequence variants

In addition to their obvious use as agonist or antagonists of therapeutically relevant molecular interactions, peptides can also be utilized as tools to study the physical nature of PPIs (102, 103). Indeed, a major advantage of using peptides for studying PPIs is that they provide a sequence-specific scaffold that can be modified with high precision. We therefore surmised that sequence variants of S1H could be used to determine which amino acid residues facilitate structural organization and biological activity of the peptide. For these studies, we employed alanine scanning to develop a library of S1H sequence variants in which individual residues within the primary sequence were replaced with alanine (Table S1). All S1H sequence variants used in this work were synthesized, purified, and characterized similarly to WT S1H as described in the Experimental procedures section. Following synthesis and purification, we evaluated the helical propensities of each variant using wavelength-dependent 10.13039/100011639CD spectropolarimetry (Fig. S7). Here, it was demonstrated that select amino acids within the S1H peptide were critical for imparting helical propensity, whereas others had little influence over the ability for the peptide to fold. Interestingly, some variants displayed >10% helicity even in the absence of TFE, indicating that the original amino acid has structure-disrupting effects. Specifically, the K39A variant exhibited 23.4% helicity in the absence of TFE, suggesting that K39 is helix-disrupting (WT S1H was only 1.1% helical under similar conditions). Other residues that were found to be helix disrupters in the S1H sequence were Y43, S44, and F45. Each of these alanine substitutions resulted in peptides that had greater than 10% helical character in the absence of TFE (Table S2). Residues that did not have a measurable impact on S1H helicity included E40, K42, and N48. As expected, all S1H sequence variants showed modest to strong increases in helicity when incubated with TFE, indicating that each construct was able to adopt a more organized fold in the presence of structure-inducing co-solvents regardless of amino acid substitution. However, peptide variants F45A and N48A each displayed minimal change in helicity following the addition of TFE, suggesting that these constructs resist folding into a more pronounced helix compared to S1H.

### Biological activity of S1H peptide sequence variants

**Figure 6:**
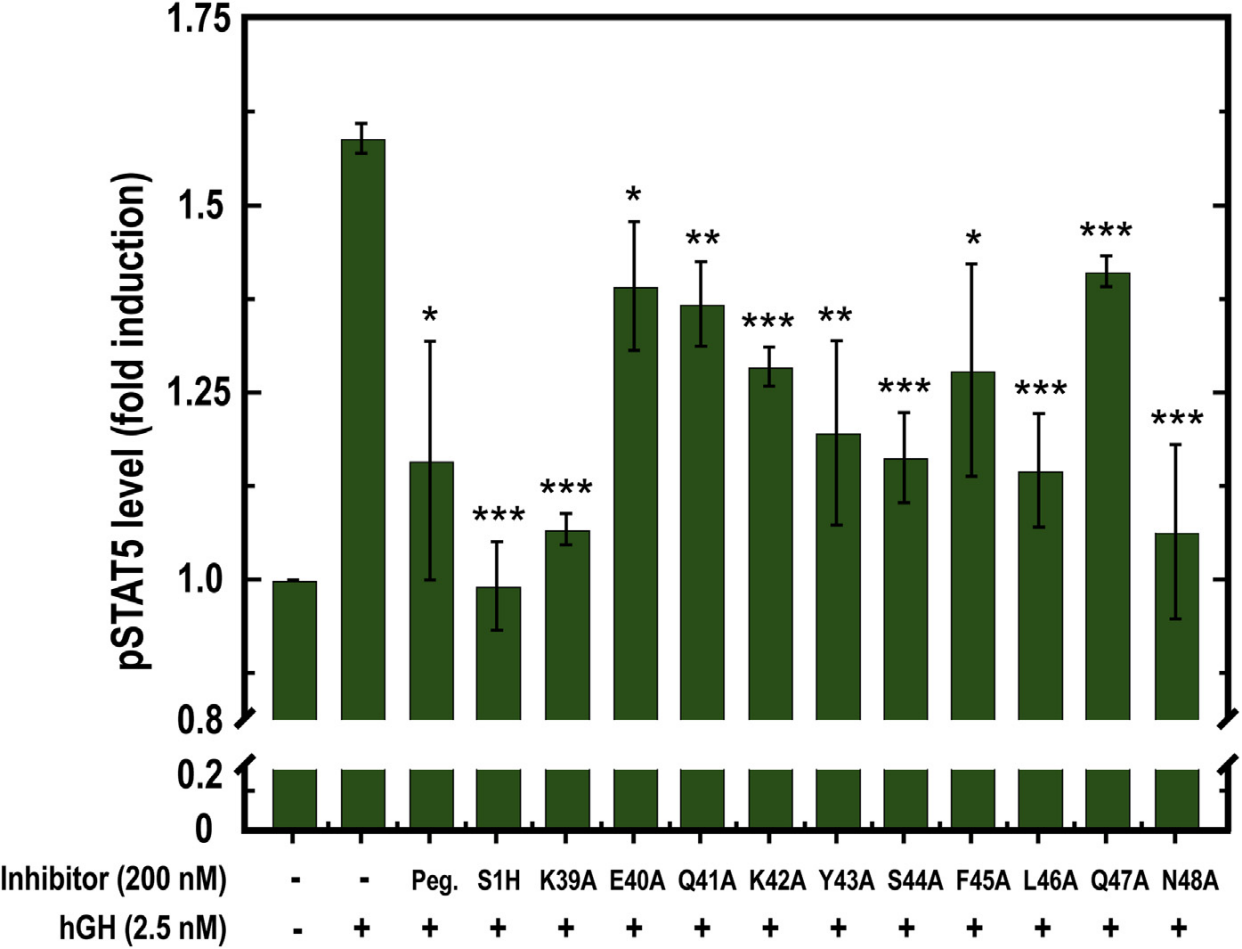
**Quantification of intracellular pSTAT5 concentrations in SK-MEL-28 cells by ELISA following co-treatment with human growth hormone (hGH) and various inhibitors.** Intracellular pSTAT5 levels are plotted as fold-induction compared with untreated controls. All peptide variants were treated at 200 nM. Pegvisomant (Peg.) was treated at 100 nM. Statistical significance indicates level of inhibition compared to cells treated with hGH alone. ∗*p* < 0.05, ∗∗*p* < 0.01, ∗∗∗*p* < 0.005.

Following the structural characterization of our S1H sequence variants, we tested the influence of singular alanine substitutions on S1H-mediated hGHR antagonism. For these experiments, we co-treated SK-MEL-28 cells with hGH and S1H variants and utilized ELISA to quantify changes in intracellular concentrations of pSTAT5 (Fig. 6). Notably, each alanine variant was able to lower intracellular hGH-mediated pSTAT5 to concentrations below that of cells treated with hGH alone, indicating that all constructs retained some antagonist activity. However, none of the sequence variants were able to reach the level of inhibition observed with WT S1H under these conditions. Notably, we observed a broad range of efficacy among the variants, with certain alanine substitutions eliciting significantly weaker antagonist effects than others. For example, S1H variants E40A, Q41A, K42A, F45A, and Q47A all displayed reduced inhibitory effects compared with WT S1H, indicating that these residues are important for S1H-mediated hGHR antagonism (Fig. 6, Table S2). On the other hand, the K39A, Y43A, S44A, L46A, and N48A variants all showed antagonist activity that was comparable to WT S1H, suggesting that alanine substitutions at these positions do not affect the ability of the S1H peptide to inhibit hGH-mediated pSTAT5. We initially anticipated that substituting S1H residues that make direct contact with the hGHR (Figure 2, Fig. S1) would strongly reduce the inhibitory effects of S1H. It was therefore surprising that we observed no obvious correlation between amino acid position and antagonist activity. With the exception of K42A and Q47A, the antagonist activity of S1H variants containing an alanine substitution at a residue that makes direct contact with the hGHR was only minimally affected. In addition, we noticed that certain sequence variants containing alanine substitutions at residues that point away from the presumed S1H–hGHR interface (such as E40A) lost almost all antagonist activity. This somewhat counterintuitive result prompted us to examine additional physicochemical properties that may be important for facilitating S1H-mediated hGHR antagonism. At this point, we noticed a strong correlation between peptide helicity and antagonist activity (Table S2). Upon comparing these two parameters, it was observed that nearly all S1H variants having greater than 60% helicity in the presence of TFE showed significant antagonism of pSTAT5. On the other hand, peptides with less than 60% helicity had reduced antagonist effects. For example, the K39A variant showed 67.1% helicity and 88.5% inhibition, whereas the E40A construct displayed 28.6% helicity and 33.4% inhibition. Taken together, these results imply that helical propensity may be more important for affecting S1H-mediated hGHR antagonism than individual side chain contacts between the peptide and receptor, at least in the context of alanine substitutions. It should be noted that we did observe two exceptions to these findings with S1H variants Q47A and N48A. Under these conditions, the Q47A peptide showed only modest (30%) inhibition despite having enhanced helical propensity (86.3%). Alternatively, the N48A variant was able to reduce pSTAT5 levels to near background (89%) levels despite having diminished helical character (12.2%). These results suggest that Q47 is important for hGHR recognition by S1H but is not needed for structural organization and that N48 is important for imparting helicity to the S1H peptide but is not required for S1H-mediated hGHR antagonism.

## Discussion

The ability to target PPIs with high precision is arguably one of the most pressing challenges facing modern medicinal chemistry. Nevertheless, the development of such molecules affords tremendous opportunities for advancing clinical research. For example, synthetic molecules that target large, shallow protein interaction surfaces not only hold considerable promise as potential therapeutics but may also be developed as chemical tools that can provide insight into the nature of biomolecular interactions. These constructs can also act as chemical genetic agents (104) that serve to expand the pool of “druggable” proteins. In this report, we developed a peptide-based hGHR antagonist (S1H) by focusing on a short, helical region of hGH that makes contact with one monomeric subunit of the hGHR homodimer. Herein, we demonstrated that the S1H peptide can fold into an α-helix in the presence of structure inducing co-solvents and also showed that it is highly stable in human serum for up to 48 h. These findings are significant as they indicate that S1H peptides have the propensity to fold into structures that mimic the site 1 mini-helix (residues 38–47) of hGH and will remain intact as they are delivered to cells in culture. Results from our stability studies also showed that the S1H peptide is more stable in human serum than pegvisomant, an FDA-approved hGHR antagonist used in the treatment of acromegaly. We attribute this somewhat surprising finding to pegvisomant possibly losing PEGylations or binding to serum proteins (91, 92) during the incubation period.

As a proof-of-principle, we demonstrated that S1H attenuates hGH-induced pSTAT5 in a dose-dependent manner in cultured cells co-treated with S1H and hGH. Owing to its structural similarity to the site 1 mini-helix contained within the “large loop” of hGH, it is likely that S1H antagonizes the hGHR by directly interfering with the hGH–hGHR interaction through site 1. To the best of our knowledge, this region of the hGHR has not yet been targeted by small molecules or other peptide-based antagonists. Therefore, the results outlined in this study serve to expand the number of potentially “druggable” sites on the hGHR. We also determined that S1H can attenuate the activation of hPRLR by showing that S1H inhibits the hPRLR-mediated phosphorylation of STAT5 in cultured cells. It has been reported previously that several amino acids of the hGH site 1 mini-helix are required for favorable hGH–hPRLR interactions (105). Moreover, significant structural deviations arise in this region when the hGH is bound to either hGHR or hPRLR (54, 106). In the context of our studies, it can be postulated that the S1H peptide interferes with ligand binding to the hPRLR, and that S1H is an effective inhibitor of lactogenic and somatotropic actions of hGH. While this conclusion obviously requires further validation, this intriguing finding suggests that S1H may be useful for inhibiting the many physiological actions of hGH *in vivo*. Indeed, this effect will become especially relevant when developing S1H as a potential therapeutic that can be used to treat hGH- or hPRL-mediated disease, including certain type of cancers.

In addition to its biological effects, the S1H peptide proved to be a useful chemical tool to study the molecular nature of the hGH–hGHR interaction. Here, we were able to use alanine scanning to identify which specific residues within the S1H sequence are important for helical propensity and biological activity. Surprisingly, substituting alanine for residues that participate in the presumed S1H–hGHR interface did not always translate to reduced antagonist activity. This finding suggests that at least in the context of alanine substitutions, many of the individual residues within the site 1 mini-helix of hGH are not explicitly required for favorable hGH–hGHR interactions. We did, however, observe a strong correlation between biological activity and helical propensity of our S1H sequence variants, which indicates that structural organization of the hGH site 1 mini-helix is important when affecting hGH binding to the hGHR. Taken together, these findings seem to suggest that the ability to form a helix is more important than amino acid sequence when modulating interactions between the hGH site 1 mini-helix and the hGHR.

Finally, it has not escaped our notice that there exists a high degree of sequence overlap between the S1H peptide and the deleted 15-residues of the 20-kDa hGH isoform. It has been shown previously that both the 22 kDa and 20 kDa hGH isoforms bind and activate the hGHR with similar efficiency (107). However, the 20 kDa hGH variant demonstrates a 10-fold weaker complex with hGHR at the site 1-binding interface compared with the 22 kDa hGH isoform. This occurs despite the 20 kDa version having a stronger (10-fold) affinity for binding site 2 compared with the 22 kDa version (82). In addition, the 20 kDa hGH isoform was found to form a 1:2 complex with the hGHR with a binding affinity that is similar to the 22 kDa hGH, but the 20 kDa hGH had markedly lower affinity to the hGHR when forming a 1:1 complex. This lower binding affinity was attributed to the deletion of the 15 amino acids, which effectively removes a major portion of the site 1 interaction domain (108). There is also considerable variation among such hGH isoforms when binding and activating the hPRLR (51). For example, the 20 kDa hGH isoform shows weaker (10-fold) activity when activating the PRLR compared with the 22 kDa isoform, indicating that residues 36 to 51 are important for hGH-mediated cross-activation of human PRLR. This observation, coupled with the results presented herein, has allowed us to speculate that S1H can antagonize hGH-mediated PRLR activation. To be sure, it remains to be seen whether S1H will inhibit the binding of the 20 kDa hGH to the hGHR with the same efficacy as it does the 22 kDa hGH. However, we anticipate that S1H binds the hGHR at a 1:1 stoichiometric ratio and will result in the competitive inhibition of both hGH isoforms.

## Experimental procedures

### Reagents and chemicals

All Fmoc-protected amino acids, Fmoc-PAL-AM resin, and PyClock were purchased from Novabiochem. Piperidine, N-methylmorpholine N,N-diisopropyl-ethylamine, trichloroacetic acid, trypsin (bovine pancreas), sodium phosphate monobasic monohydrate, glycerol, triisopropylsilane, N-methyl-2-pyrrolidone (NMP), RIPA lysis buffer, and Bradford reagent were purchased from Sigma-Aldrich. Acetonitrile (ACN) was purchased from Alfa Aesar. β-mercaptoethanol was purchased from Aldrich Chemicals. Potassium chloride was obtained from VWR Analytical. Human AB serum was purchased from Corning. Cell lines: SK-MEL-28, MALME-3M, IM-9, T-84, mouse fibroblasts (L), growth medium: Dulbecco's modified Eagle's medium, Eagle's Minimal Essential Medium, RPMI-1640, and fetal bovine serum were each purchased from American Type Culture Collection (ATCC). 2,2,2-trifluoroethanol, acetic acid, dichloromethane, trifluoroacetic acid (TFA), phenol, Tris-base, Tris-HCl, calcium chloride, sodium chloride, potassium dihydrogen phosphate, sodium dodecyl sulfate, antibiotic-antimycotic, Halt protease, phosphatase inhibitor cocktail, and chemiluminescence detection reagents were purchased from Thermo Fisher Scientific. Recombinant hGH was obtained from Antibodies Online. Recombinant (active) human prolactin/PRL protein was obtained from Abcam. Hydroxybenzotrizaole was purchased from Apex Bio. All other materials were purchased from commercial sources and used without further purification.

### Peptide synthesis

All peptides described herein were synthesized on PAL-AM resin using standard Fmoc solid-phase peptide synthesis procedures (109) Solid-phase peptide synthesis reactions were performed on a 25 μmol scale in fritted glass reaction vessels to facilitate removal of reactants and starting materials. All amino acid couplings and Fmoc deprotections were performed a microwave-accelerated reaction system (CEM) using software programs written in-house. Multiple rounds of washing using fresh NMP were performed between each coupling and deprotection step described below. N-terminal amino acid couplings were achieved by treating the deprotected peptide-resin with five equivalents (eq) of Fmoc-protected amino acid, 5 eq PyClock, and 10 eq of N-methylmorpholine N,N-diisopropyl-ethylamine in NMP. All equivalents were based on resin loading level. N-terminal Fmoc groups were removed by treating the peptide-resin with 25% (v/v) piperidine containing 0.1 M hydroxybenzotrizaole to minimize aspartimide formation (110). Iterative cycles of amino acid coupling and deprotections were repeated until peptides of the desired sequence were obtained. Following synthesis, the peptides were capped by treating the deprotected peptide-resin with 6% (v/v) acetic anhydride and 6% (v/v) N-methylmorpholine in NMP for 20 min at room temperature. This step was repeated once, and the acylated peptide resin was washed thoroughly with NMP to remove any unreacted starting materials. Following the final wash, the peptide resin was rinsed with NMP and dichloromethane and dried under vacuum to remove residual solvent.

### Cleavage and purification of peptides

Following completion of the synthesis, resin-bound peptides were globally deprotected and cleaved from the resin by adding a cleavage cocktail composed of TFA, water, phenol, and triisopropylsilane (85:5:5:2, v/v/v/v). The cleavage reaction was allowed to incubate for 30 min at 38 °C in a microwave reactor (CEM). Following completion of the cleavage reaction, the peptides were precipitated in cold diethyl ether, pelleted by centrifugation, and resuspended in aqueous ACN (15%, v/v). This solution was then frozen and lyophilized to remove residual solvent. Following lyophilization, crude peptide powders were resuspended in aqueous ACN (15%, v/v) and purified across a C18 reversed-phase HPLC column (Grace, 10 μm, 250 × 10 mm) using a ProStar HPLC system (Agilent). Peptides were eluted over 30 min with a linear gradient of 15 to 45% solvent B (0.1% TFA in ACN) over solvent A (0.1% TFA in water). Absorbance spectra were monitored at 214 nm and 280 nm to distinguish peptide products, and all major peaks were evaluated by mass spectrometry to determine peptide identity. Following mass analysis, fractions containing desired peptides were combined, frozen, and lyophilized twice. Peptide stock solutions were made by dissolving the purified peptides in water and storing them at 4 °C protected from light. All peptide concentrations were quantified using extinction coefficients calculated based on their primary sequences. Peptides were found to be stable for at least 1 year under these storage conditions.

### General characterization of peptides by electrospray ionization mass spectrometry and analytical RP-HPLC

All peptide products were identified using electrospray ionization mass spectrometry (Fig. S2 and Table S1). Masses were collected using a Q Exactive Plus Hybrid Quadrupole-Orbitrap mass spectrometer (Thermo Scientific) in the range of 500 to 2200 *m/z*. For mass analysis, peptides were dissolved in a suitable volume of aqueous ACN (10%, v/v) and directly injected onto the electrospray ionization system. Mass data were processed using Xcalibur v3.0 (Thermo) and MagTran v1.0 deconvolution software (Amgen). Peptide purities were determined by analytical reversed-phase HPLC using an Agilent ProStar system. For analysis, peptides were dissolved in water at a final concentration of 2.5 μM and injected across a reversed-phase C18 column (Thermo, 5 μm, 50 × 2.1 mm). Peptides were eluted over 20 min with a linear gradient of 5 to 95% solvent B (0.1% TFA in ACN) over solvent A (0.1% TFA in water). All peptides were purified to >95% as determined by product peak integration of analytical HPLC chromatograms (Fig. S2). Analytical HPLC data were processed using OpenLab CDS ChemStation Software (Agilent) v1.06 and KaleidaGraph v4.5 (Synergy Software).

### Structural characterization of peptide oligomers

The solution-phase structure of each peptide was evaluated by wavelength-dependent CD spectropolarimetry. For analysis, stock peptides were diluted to a final concentration of 20 μM in PBS supplemented with or without 30% (v/v) TFE. All peptide solutions were allowed to equilibrate for 10 min at 25 °C before analysis. Far-UV wavelength scans were performed on a Jasco J-715 CD spectropolarimeter from 255 nm to 195 nm at 25 °C. Final spectra were generated from a background subtracted (buffer only) average of four scans. Data were processed using J-700 Software v1.5 (Jasco) and KaleidaGraph v4.5. Percent helicity was calculated from the mean residue ellipticity at 222 nm using Equation 1:

(1) $$\%\text{helical content}=100\;\times \;\left({\left[\text{θ}\right]}_{222}/\left(-39,000\left(1-2.57/n\right)\right)\right)$$

where [θ]_222_ is the mean residue ellipticity at 222 nm and *n* is the total number of peptide bonds (86).

### *In vitro* stability of S1H peptide

Serum stability tests for S1H were performed by dissolving the peptide at a final concentration of 51.6 μg/ml in 200 ml prewarmed RPMI medium supplemented with 25% (v/v) heat-inactivated human AB serum. The solutions were then allowed to incubate at 37 °C for 0, 1, 4, 12, 24, or 48 h. Following incubation, 400 μl of 15% (w/v) trichloroacetic acid in water was added to each tube, and the mixtures were quickly cooled on ice for 15 min. The samples were then centrifuged three times at 14,000 rpm to remove precipitated proteins. Once centrifuged, 400 μl of the supernatant was removed and directly injected onto a C18 reversed-phase HPLC column (Thermo, 5 μm, 50 × 2.1 mm). The peptides were resolved using a linear gradient of 0 to 50% solvent B over solvent A in 20 min. Fraction of intact peptide was quantified from product peak integration and normalized to undigested controls. As positive controls for peptide degradation, S1H was dissolved at a final concentration of 34.4 μg/ml in 100 μl trypsin digestion buffer (100 mM Tris-base, 1 mM CaCl_2_, pH 7.8) containing 10 μg/ml trypsin. This reaction was allowed to incubate at 37 °C for 15 min. Following incubation, 100 μl of 50% (v/v) TFA in water was added, and the reactions were quickly cooled on ice for 15 min. The samples were then centrifuged three times at 14,000 rpm to remove precipitated proteins, and 150 μl of the supernatant was removed for analysis by HPLC. The sample was directly injected onto a C18 reversed-phase column (Thermo, 5 μm, 50 × 2.1 mm) and resolved using a linear gradient of 0 to 50% solvent B over solvent A in 50 min.

### *In vitro* stability of hGH and pegvisomant

Stability tests for recombinant proteins were performed by dissolving hGH or pegvisomant at a final concentration of 25 μg/ml in either 450 ml (for hGH) or 600 ml (for pegvisomant) prewarmed RPMI medium supplemented with 25% (v/v) heat-inactivated human AB serum. These solutions were then allowed to incubate at 37 °C for 0, 1, 4, 12, 24, or 48 h. For Western blot analysis, 60 μl (for hGH) or 80 μl (for pegvisomant) of each reaction solution were removed at the indicated times and mixed with 5× Laemmli loading buffer. The samples were then incubated at 95 °C for 5 min before being cooled on ice and stored at −80 °C. Once all samples were prepared, 25 μl of each protein solution were loaded onto polyacrylamide gels (12% for hGH and 8% for pegvisomant) and separated by SDS-PAGE. Following separation, the proteins were transferred to polyvinylidine difluoride membranes and subjected to Western blot analysis (*vide infra*). The fraction of intact protein was quantified by measuring the integrated density of each band contained within regions of the gel corresponding to “intact” or “degraded” proteins. As positive controls for hGH and pegvisomant degradation, the proteins were dissolved at a final concentration of 25 μg/ml in 60 μl (for hGH) and 80 μl (for pegvisomant) trypsin digestion buffer containing 4 μg/ml trypsin. These reactions were allowed to incubate at 37 °C for up to 8 h, with samples removed at selected timepoints for analysis. Following incubation, the samples were either mixed with 5× Laemmli loading buffer and subjected to SDS-PAGE for Western blot analysis (Fig. S5) or directly injected onto a C18 reversed-phase column for analysis by HPLC as described above for the S1H peptide (Fig. S4).

### Cell culture and hGH treatment

SK-MEL-28, MALME-3M, and T84 cells were grown and maintained in Eagle's Minimal Essential Medium. IM9 and L cells were grown in RPMI-1640 media and Dulbecco's modified Eagle's medium, respectively. All growth media was supplemented with 10% fetal bovine serum and 1× antibiotic-antimycotic (penicillin-streptomycin) solution. Cells were grown at 37 °C under a 5% CO_2_ atmosphere in a humidified incubator, and growth media was replaced every 48 h during incubation. No hGH was present in or added to the growth media unless specifically indicated. Between 12 to 16 h after seeding, adherent cells (SK-MEL-28, MALME-3M, T84, L cells) were switched to serum-free growth media (no GH) and starved for 4 h before the addition of hGH, bGH (L cells), hPRL (MALME-3M and T84 cells), or buffer at the concentrations noted. Cells were then allowed to incubate for an additional 20 min before being harvested. For suspended cell treatments, IM9 cells were pelleted by centrifugation at 140*g* for 5 min at room temperature. The supernatant was then removed by aspiration, and the cells were resuspended in serum-free media. The cells were then starved for 4 h at 37 °C under a 5% CO_2_ atmosphere in a humidified incubator and subsequently treated with hGH, S1H, or both at the indicated concentrations for 20 min before harvesting by centrifugation.

### Protein extraction

Following treatment, the cells were harvested at the indicated time points, and the total protein was extracted. To affect protein extraction, the treatment media was removed by aspiration, and the cells were washed twice with ice-cold PBS. Following washing, total protein was extracted from the cells using RIPA buffer (100 μl/million cells) supplemented with 1.5× Halt protease and phosphatase inhibitor cocktail. Briefly, chilled RIPA buffer was added to the cells, and the lysis reaction was allowed to incubate for 5 min at 4 °C. Adherent cells were harvested for lysis using a sterile cell scraper. The resultant cell suspensions were sonicated on ice for 60 s with ON (2-s)/OFF (1-s) pulses at 2% power. The cell lysate was then cleared by centrifugation at 8000*g* for 10 min at 4 °C. Following centrifugation, the supernatant was collected and stored at −80 °C until further use. Sample protein concentration was quantified using a Bradford assay with bovine serum albumin as the protein standard. Absorbance at 595 nm was measured using a Spectramax 250 multimode plate reader (Molecular Devices) and processed using SoftmaxPro v4.7.1 software.

### Western blotting

Cytosolic fractions from treated cells were thawed on ice, and 80 μg protein was loaded onto polyacrylamide gels for separation by SDS-PAGE. All SDS-PAGE gels were resolved at 120 V for 1.5 h at 20 °C. Following separation, the proteins were transferred to activated polyvinylidine difluoride membranes using a wet-transfer method at 20 V for 16 h at 4 °C. The membrane was then blocked with 5% bovine serum albumin in 1× TBS-T (Tris buffered saline, 0.1% Triton-X100, pH 7.2) for 2 h at 25 °C and incubated with primary antibody at specified dilutions for 16 h at 4 °C. Following addition of primary antibody, the membrane was washed and subsequently incubated with appropriate secondary antibodies at the indicated dilutions for 2 h at 25 °C. Membranes were then washed with TBS-T and treated with West Femto Chemiluminescence detection reagents (Thermo Fisher Scientific). Chemiluminescence signals were detected using a GelDoc (BioRad) fluorescence imager. Densitometry analyses of the blots was performed using ImageJ Software (111) by measuring the integrated band density and normalizing against band intensity of the loading control (β-actin) in the same lane. The integrated densities of pSTAT5 bands from cells after different treatments were then normalized to integrated densities of pSTAT5 bands from cells treated with hGH alone to obtain an induction ratio. Percent inhibition was then measured by subtracting the pSTAT5 band intensity in each lane from the pSTAT5 band intensity of hGH-treated samples. A list of antibodies, commercial sources, and dilution ratios is provided in Table S3.

### ELISA assay

Intracellular pSTAT5A (Y694) and pSTAT5B (Y699) levels were quantified from clarified protein samples using an InstantOne ELISA kit (Thermo Fisher) according to the manufacturer’s instructions. Briefly, 50 μl of protein sample (1 mg/ml) were added to wells containing 50 μl antibody cocktail in a 96-well plate and incubated at room temperature for 2 h with shaking (300 rpm). Following incubation, the wells were washed, and the bound antibodies were detected by adding detection regent and incubating the plate for 1 h at room temperature with shaking (300 rpm). Following incubation, stop reagent was added, and the absorbance was read at 450 nm using a UV-VIS spectrophotometer (Molecular Devices, model M2). All experiments were performed in triplicate. Percent pSTAT5 inhibition was calculated from background-subtracted pSTAT5 levels using Equation 2:

(2) $$\%\text{inhibition}=100\;\times \;\left({\text{pSTAT}5}_{\text{hGH}}-{\text{pSTAT}5}_{\text{PI}}\right)/{\text{pSTAT}5}_{\text{hGH}}$$

where pSTAT5_hGH_ is the fold-induction of pSTAT5 upon treatment with hGH alone and pSTAT5_PI_ is the fold-induction of pSTAT5 upon co-treatment with hGH and peptide inhibitor.

### Statistical analysis

All data points represent an average of three independent experiments; error bars are plotted as standard deviations. A two-tailed Student’s *t* test was used to analyze statistical significance after testing for distribution, and a 95% confidence interval was used to denote the significance.

## Data availability

All data described in the manuscript are contained in the main text or supporting information.

## Supporting information

This article contains supporting information.

## Conflict of interest

The authors declare that they have no conflicts of interest with the contents of this article.
