# Supplementary material for: A novel peptide antagonist of the human growth hormone receptor
Source: J Biol Chem. 2021 Mar 24;296:100588. doi: 10.1016/j.jbc.2021.100588 (PMC8086144; doi:10.1016/j.jbc.2021.100588)
Supplement: Figures S1 to S7 and Table S1 [file mmc1.pdf]

Supporting Information

A novel peptide antagonist of the human growth hormone receptor

Reetobrata Basu[1,2], Khairun Nahar[3], Prateek Kulkarni[2,4], Olivia Kerekes[3], Maya Sattler[3,5], Zachary Hall[3], Sebastian Neggers[6], Justin M. Holub[2,3,4,*] and John J. Kopchick[1,2,4,*]

[1]Heritage College of Osteopathic Medicine, [2]Edison Biotechnology Institute, [3]Department of Chemistry and Biochemistry, [4]Molecular and Cellular Biology Program, [5]Honors Tutorial College, Ohio University, Athens, OH, 45701, USA. [6]Department of Medicine, Endocrinology Section, Erasmus University Medical Centre, Rotterdam, The Netherlands.

Peptide	Sequence	Mass data	
		Calc. (+m/z)	Obs. (+m/z)
S1H (36-51)	^{Ac}YIPKEQKYSFLQNPQT-NH_2	2025.26	2026.04
S1H (K39A)	^{Ac}YIPAEQKYSFLQNPQT-NH_2	1968.99	1968.99
S1H (E40A)	^{Ac}YIPKAQKYSFLQNPQT-NH_2	1967.23	1967.03
S1H (Q41A)	^{Ac}YIPKEAKYSFLQNPQT-NH_2	1968.22	1968.02
S1H (K42A)	^{Ac}YIPKEQAYSFLQNPQT-NH_2	1967.18	1967.98
S1H (Y43A)	^{Ac}YIPKEQKASFLQNPQT-NH_2	1933.17	1933.02
S1H (S44A)	^{Ac}YIPKEQKYAFLQNPQT-NH_2	2010.50	2010.10
S1H (F45A)	^{Ac}YIPKEQKYSALQNPQT-NH_2	1950.15	1950.00
S1H (L46A)	^{Ac}YIPKEQKYSFAQNPQT-NH_2	1983.19	1983.00
S1H (Q47A)	^{Ac}YIPKEQKYSFLANPQT-NH_2	1968.22	1968.02
S1H (N48A)	^{Ac}YIPKEQKYSFLQAPQT-NH_2	1983.10	1983.00

Table S1. Sequence and mass data of peptides used in this work. Substituted alanine residues are shown in red. Calc.: calculated mass (+*m/z*); Obs.: observed mass (+*m/z*).

Peptide	$[\theta]_{222}$		% α-helix[a]		% pSTAT5 inhibition[b]
	- TFE	+ TFE	- TFE	+ TFE	
S1H (36-51)	-400	-16,000	1.1	48.2	98.3
S1H (K39A)	-7,800	-22,300	23.4	67.1	88.5
S1H (E40A)	-1,900	-9,500	5.7	28.6	33.4
S1H (Q41A)	-2,700	-8,400	8.3	25.4	37.4
S1H (K42A)	-800	-8,100	2.4	24.4	51.7
S1H (Y43A)	-3,700	-22,800	11.2	68.8	66.6
S1H (S44A)	-5,200	-21,100	15.7	63.5	72.3
S1H (F45A)	-3,700	-7,100	11.1	21.4	52.5
S1H (L46A)	-2,300	-28,600	7.0	86.3	75.1
S1H (Q47A)	-2,700	-28,600	8.2	86.3	30.1
S1H (N48A)	-2,200	-4,100	6.7	12.2	89.1

Table S2. Structural data and biological activity of peptides used in this work. [a]Percent helicity was calculated from the background-subtracted mean residue ellipticity (MRE) at 222 nm. [b]Percent pSTAT5 inhibition was calculated from pSTAT5 levels obtained from ELISA experiments. See Materials and Methods section of main text for details.

#	Antibody Target	Host	Vendor	Cat. #	Dilution
1	phospho-STAT5B (Y699)+STAT5B (Y694)	Rabbit	RnD Biosystems	MAB41901	1:500
2	Total STAT5	Rabbit	Cell Signaling Technology	9358S	1:1000
3	Beta-Actin	Rabbit	Cell Signaling Technology	4970S	1:4000
4	Anti-Rabbit HRP conjugated	Goat	Invitrogen	65-6120	1:10000
5	Anti-GH antibody	Rabbit	Abcam	ab51257	1:2000
6	Anti-PEG antibody	Rabbit	Abcam	ab155276	1:10000

Table S3. Details of primary and secondary antibodies used in this work.

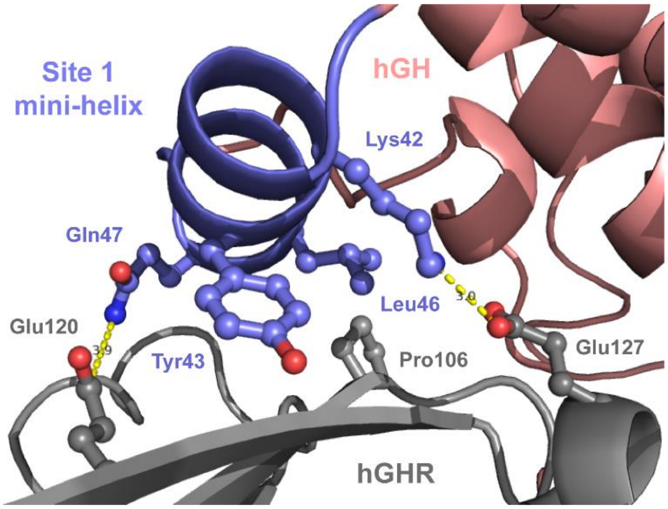

Figure S1: Binding between the hGH site 1 mini-helix (residues 38-47) and the hGHR is facilitated through various types of interactions, including hydrogen bonding, hydrophobic effects and electrostatic interactions. Hydrogen bonding pairs include Lys42-Glu127 and Gln47-Glu120, with distances shown as dotted yellow lines. Both proteins are rendered as ribbon diagrams with interacting side-chains shown as ball-and-stick. The hGH is colored salmon, the hGH site 1 mini-helix is colored blue and the hGHR is grey. Image adapted from PDB ID: IHWG; figure rendered in PyMol.

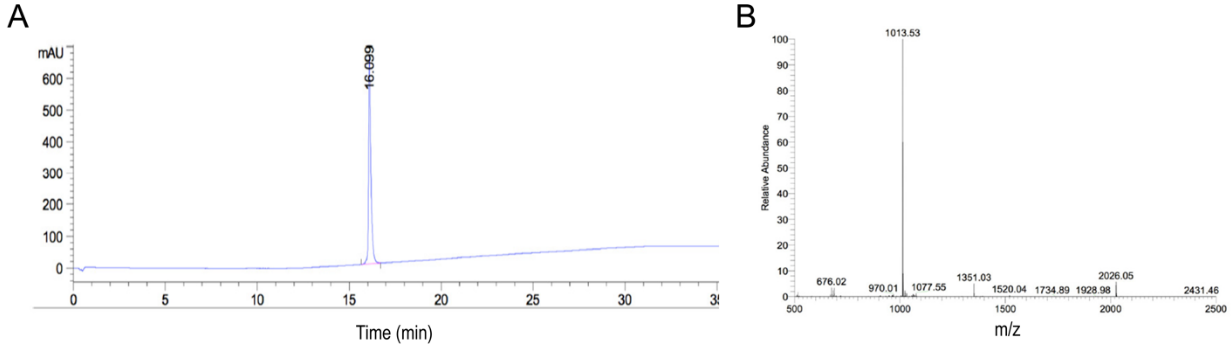

Figure S2. (A) Analytical RP-HPLC chromatogram of S1H peptide. Elution was monitored at 214 nM. AU: absorbance units. B) Mass spectrum of S1H peptide; calculated mass: 2025.26 m/z; observed mass: 2026.05 m/z. Prominent half-mass ionization peak at 1013.53 m/z is shown with 100% relative abundance.

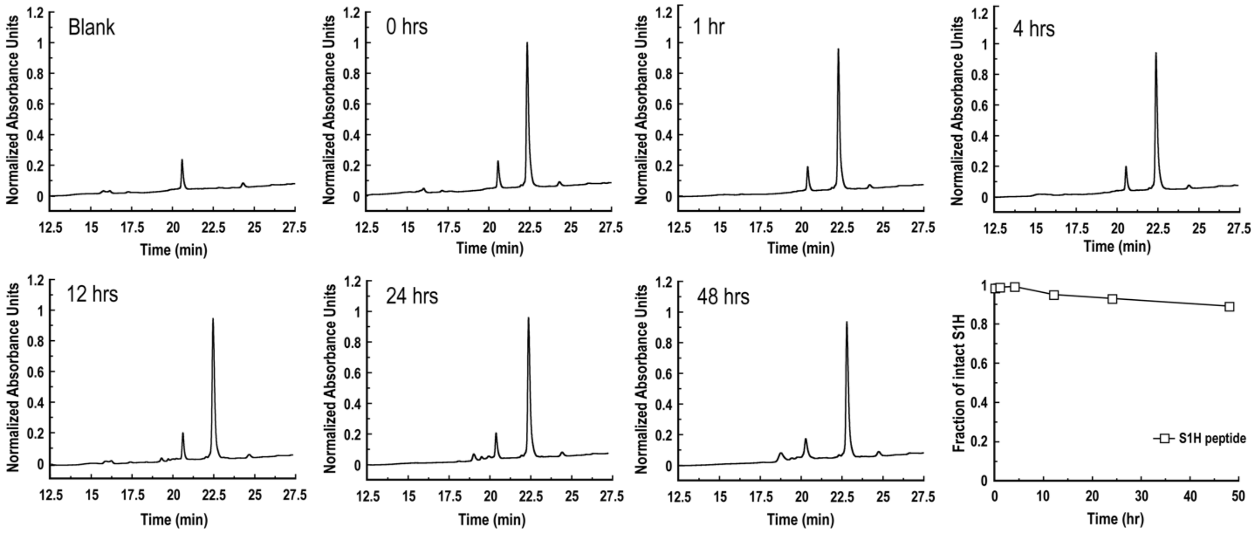

Figure S3. *In vitro* stability of S1H peptide incubated in RPMI media supplemented with 25% (v/v) human AB serum as analyzed by analytical RP-HPLC. Incubation times are shown at the top left of each chromatogram. All spectra were recorded at 214 nm and show traces normalized to the 0 h chromatogram. Bottom right panel shows line graph of intact peptide as determined from product peak integration of each spectra at respective timepoints. The small peaks present at 16, 20.5 and 24 min in each chromatogram represent background (blank) serum proteins and were not included in the final integration calculations.

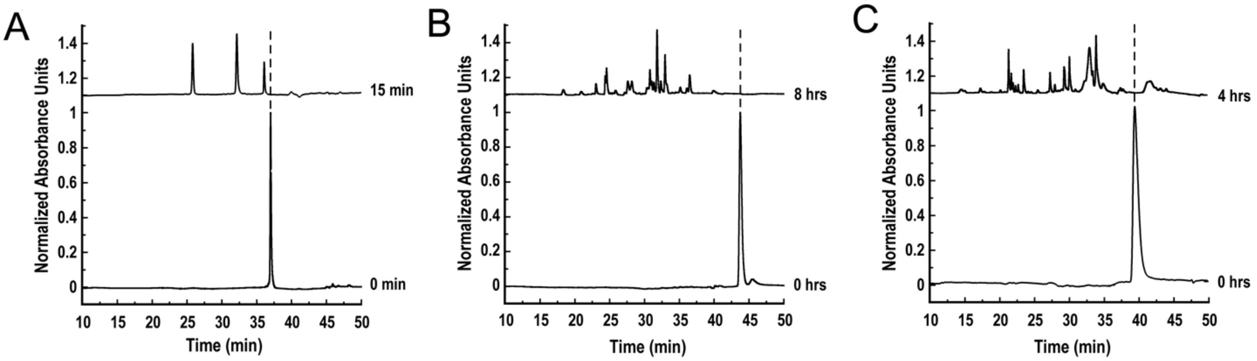

Figure S4. *In vitro* stability of peptides and proteins incubated with trypsin as analyzed by reversed-phase HPLC. Lower spectra show HPLC traces of peptides and proteins alone; upper spectra show HPLC traces of peptides and proteins incubated with trypsin for the indicated timepoints; upper spectra is offset by 1.1 normalized absorbance units. All spectra were recorded at 214 nm and show traces normalized to the untreated controls. (A) S1H peptide; (B) human growth hormone; (C) pegvisomant.

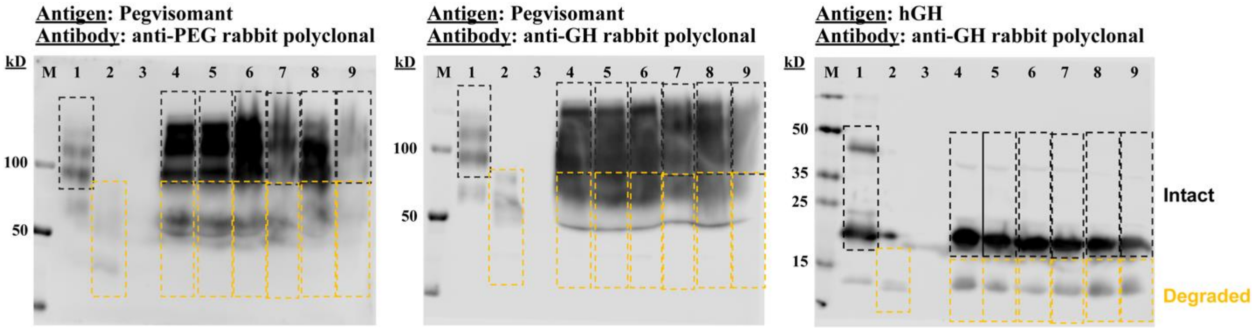

Figure S5. *In vitro* stability of hGH and pegvisomant incubated in RPMI media supplemented with 25% (v/v) human AB serum as analyzed by western blot. Test protein (antigen) and primary antibody used for blotting are indicated above each panel. Size marker (kD) is shown to the left of each blot. Lane designation: M: marker; 1: fully intact protein (no incubation); 2: degraded protein (trypsin digestion); 3: blank sample (no protein); 4: 0 h incubation; 5: 1 h incubation; 6: 4 h incubation; 7: 12 h incubation; 8: 24 h incubation; 9: 48 h incubation. Regions of the gel used for densitometry analysis are shown in black (for intact protein) and orange (for degraded protein).

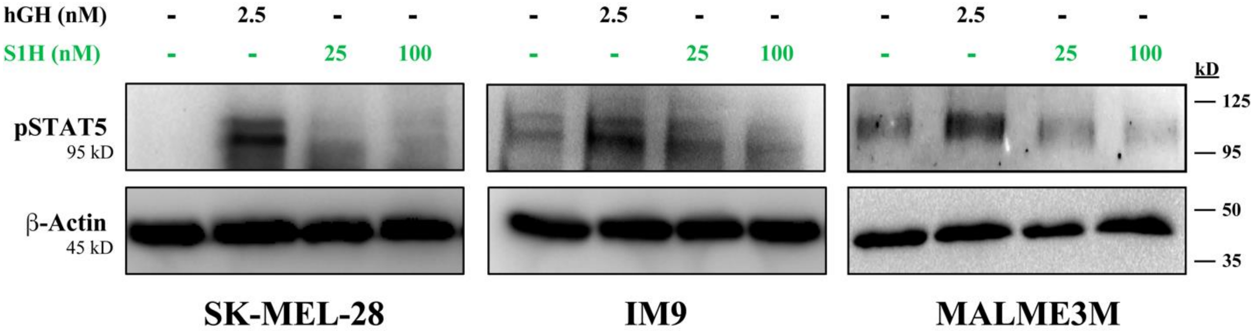

Figure S6. The S1H peptide does not increase pSTAT5 levels in cultured SK-MEL-28, MALME3M or IM9 cells at concentrations up to 100 nM following 20 min incubation. Size marker (kD) is shown to the right of the images. See Materials and Methods of the main text for experimental details.

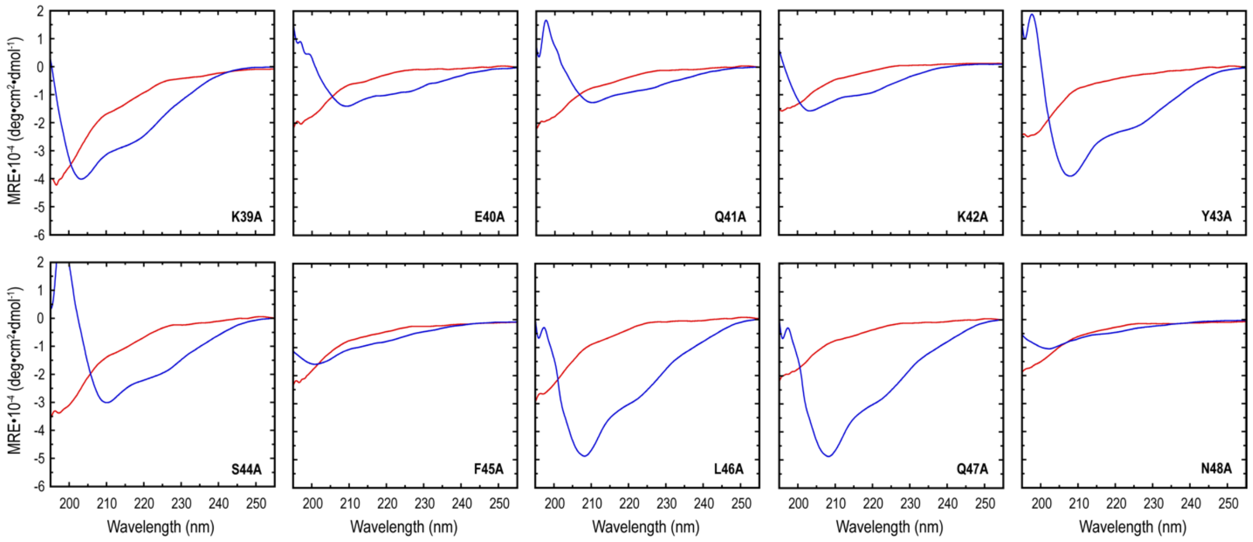

Figure S7. Wavelength-dependent circular dichroism spectra of S1H sequence variants (20 μM) in PBS. Red spectra show S1H sequence variants in PBS and blue spectra represent the same peptides in PBS supplemented with 30% (v/v) TFE. Amino acid substitution in S1H is shown in the bottom right of each spectrum. All spectra were collected from 195 nm to 255 nm at 25 ºC.
